# Research Progress and Future Perspectives of Orexin Receptor Antagonists in Insomnia Therapy

**DOI:** 10.3390/molecules31162795

**Published:** 2026-08-11

**Authors:** Zirui Zhang, Lina Zhu, Yue Gao, Caoqing Ji, Yong Ling

**Affiliations:** 1 Academy of Pharmacy, Xi’an Jiaotong-Liverpool University, Suzhou 215123, China; changzzzhr@gmail.com; 2 School of Pharmacy, Nantong University, Nantong 226001, China

**Keywords:** insomnia, orexin, orexin receptor antagonists, sleep–wake regulation, high selectivity

## Abstract

Insomnia is a common sleep disorder that significantly impairs quality of life and increases the risk of various chronic diseases. Although conventional sedative–hypnotic agents are effective, their clinical use is limited by tolerance, dependence, and residual next-day effects. The orexin system plays a critical role in sleep–wake regulation through orexin receptor 1 (OX1R) and orexin receptor 2 (OX2R), with OX2R serving as the primary mediator of wakefulness. Based on this mechanism, orexin receptor antagonists (ORAs) have been developed to promote sleep by suppressing wake-promoting signaling pathways. This review systematically summarizes the structural and functional characteristics of the orexin system, with particular emphasis on the functional divergence of OX1R and OX2R and their therapeutic relevance. Recent advances in dual orexin receptor antagonists (DORAs) and selective orexin receptor antagonists are discussed, and the pharmacological properties and clinical performance of approved and investigational agents are compared. In addition, the safety profiles, adverse effects, and clinical limitations of ORAs are reviewed. Future perspectives are highlighted, including receptor selectivity, pharmacokinetic optimization, and personalized treatment strategies. Collectively, ORAs offer a mechanism-based therapeutic approach that may improve both the efficacy and safety of insomnia treatment.

## 1. Introduction

In recent years, increasing life stress and environmental influences have contributed to sleep disturbances becoming a widespread public health concern [1]. Insomnia negatively affects daytime work performance and quality of life and is also recognized as an important risk factor for various disorders, including cardiovascular diseases, depression, and cognitive impairment [2]. Although cognitive behavioral therapy is recommended as the first-line treatment for insomnia, pharmacological intervention remains an indispensable therapeutic approach in clinical practice because of poor patient adherence and delayed therapeutic onset [3].

The frequent coexistence of insomnia and depressive symptoms further highlights the involvement of broader neuropsychiatric signaling networks. Emerging studies have identified GPR56, trace amine-associated receptor 1 (TAAR1), and vasopressin V1B receptors as potential pharmacological targets in neurological or depressive disorders [4,5]. In addition, model-informed precision dosing of sertraline with zopiclone co-administration illustrates the growing importance of individualized treatment strategies in psychiatric patients with sleep-related symptoms [6].

The development of sleep-promoting medications reflects the progressive understanding of the neurobiological mechanisms underlying sleep regulation. Early therapeutic strategies relied on barbiturates, which enhance γ-aminobutyric acid (GABA)-mediated inhibition in a non-selective manner [7,8]. Subsequently, benzodiazepines were developed to selectively modulate GABA receptors; however, they remain associated with cognitive impairment and increased fall risk [9], as well as potential disruption of natural sleep architecture [10]. Later, non-benzodiazepine sedative–hypnotics (Z-drugs) emerged, offering more controllable durations of action and relatively reduced adverse effects. Thereafter, melatonin receptor agonists targeting circadian rhythm regulation were introduced to achieve physiological sleep modulation through activation of MT1 and MT2 receptors [11,12]. In recent years, research has increasingly focused on wakefulness regulatory pathways [13], thereby promoting the development of therapeutic strategies targeting the orexin system.

Circadian disruption may also be induced by pharmacological suppression of melatonin secretion, as demonstrated in a zebrafish prednisolone model [12]. Beyond its chronobiological effects, melatonin has been reported to attenuate oxidative stress and inflammatory signaling through Sirt1 activation, indicating that melatonin-related pathways may connect circadian regulation with cellular stress responses [14].

With a deeper understanding of the neural mechanisms underlying sleep regulation, research has gradually shifted from “global suppression of wakefulness” toward “targeted modulation of wake-promoting pathways”. Among multiple wakefulness regulatory systems, the orexin system is regarded as a key regulatory network responsible for maintaining wakefulness. Orexin neurons are predominantly distributed within the lateral hypothalamus and project extensively to wake-promoting centers, including the locus coeruleus, dorsal raphe nucleus, and tuberomammillary nucleus, thereby integrating multiple neurotransmitter signals to sustain stable wakefulness [15,16]. Therefore, compared with conventional sedative–hypnotic agents that induce sleep through generalized central nervous system suppression, therapeutic strategies targeting the orexin system regulate sleep by reducing wake-promoting drive, resulting in a mechanism closer to physiological sleep processes and exhibiting notable safety advantages [17,18]. Based on this mechanism, orexin receptors have gradually emerged as important therapeutic targets for the development of novel anti-insomnia agents.

**Figure 1:**
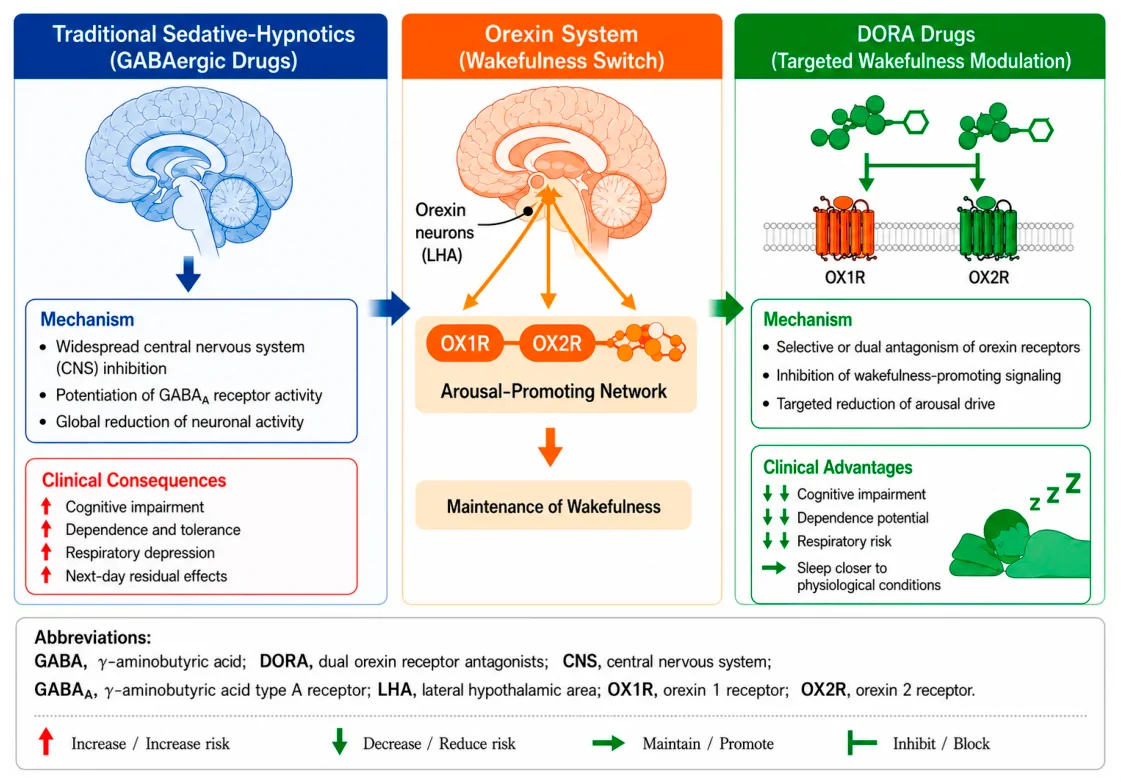
Evolution of pharmacological treatments for insomnia. The pharmacological management of insomnia has evolved from traditional GABAergic sedative–hypnotics to orexin receptor antagonists, reflecting a shift from non-selective central nervous system suppression to targeted modulation of wakefulness through the orexin system. Abbreviations: ORAs, orexin receptor antagonists; DORA, dual orexin receptor antagonist; OX1R, orexin receptor type 1; OX2R, orexin receptor type 2; LHA, lateral hypothalamic area; GABA, γ-aminobutyric acid; GABAA receptor, γ-aminobutyric acid type A receptor; CNS, central nervous system.

Several comprehensive reviews on orexin receptor antagonists have recently summarized their pharmacological mechanisms, clinical efficacy, and safety profiles in insomnia treatment. However, these reviews mainly focus on the clinical application of approved ORAs and provide relatively limited discussion of recent advances in structural biology, receptor subtype selectivity, and rational drug design. In contrast, the present review integrates recent progress in cryo-electron microscopy, receptor–ligand interactions, and structure–activity relationships to provide a more comprehensive understanding of the molecular basis of ORA function. Furthermore, this review summarizes the latest developments in both approved and investigational ORAs, including emerging selective OX2R antagonists and newly developed compounds from China, and discusses future directions from the perspectives of receptor selectivity, pharmacokinetic optimization, and precision medicine. Accordingly, this review not only summarizes current clinical evidence but also provides a structural and translational perspective that may facilitate the future development of next-generation orexin receptor antagonists [19,20,21]. The evolution of pharmacological therapies for insomnia is summarized in Figure 1.

This review aims to systematically summarize the research progress of ORAs. Beginning with the structure–activity relationships of the orexin system, the pharmacological characteristics and clinical evidence of approved DORAs are compared, their clinical value and limitations are evaluated, and future development directions of sleep therapeutics are further discussed.

## 2. The Orexin System

The orexin system, also known as hypocretin system, was independently discovered by two research groups in 1998 [22,23,24]. This system is produced by a relatively small population of neurons located in the lateral hypothalamus; however, their axons project extensively to multiple arousal-related nuclei throughout the central nervous system, where they play a critical role in maintaining wakefulness and regulating sleep–wake rhythms [15]. The following sections discuss the structural and functional characteristics of orexins and orexin receptors, followed by an overview of orexin receptor antagonists.

### 2.1. Orexin Receptors

#### 2.1.1. Structural Characteristics of Orexin Receptors

**Figure 2:**
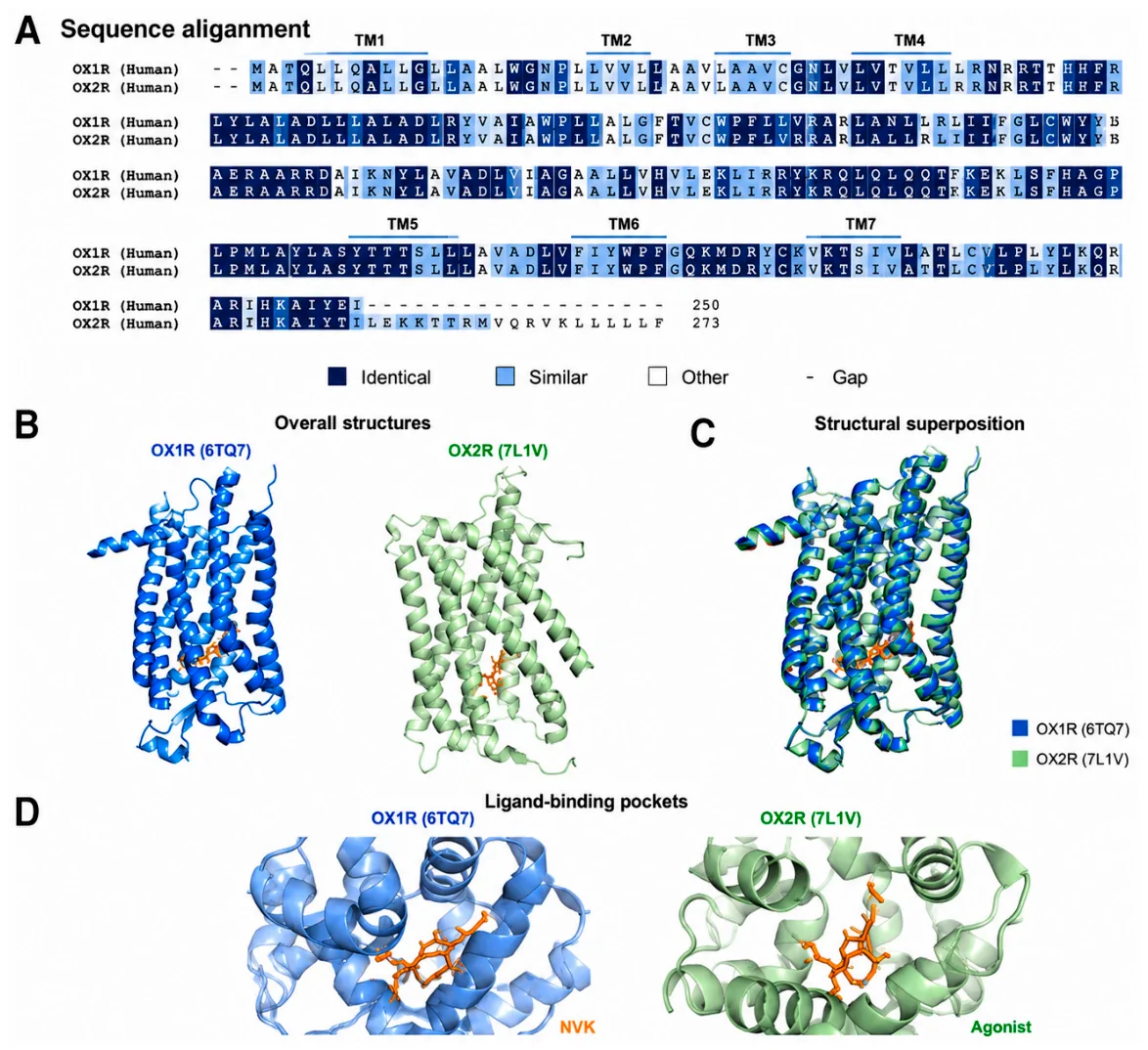
Structural comparison of human orexin receptors. (**A**) Sequence alignment of human OX1R and OX2R highlighting conserved transmembrane regions and sequence similarity. (**B**) Overall structures of human OX1R (PDB ID: 6TQ7) and OX2bR (PDB ID: 7L1V). (**C**) Structural superposition of OX1R and OX2R. (**D**) Comparison of ligand-binding pockets showing representative bound ligands. The structural models shown in this figure were prepared using the experimentally determined structures reported by Rappas et al. [34] and Hong et al. [35].

Orexin receptors are membrane-bound receptors that mediate orexin signaling and belong to the class A (rhodopsin-like) G protein-coupled receptor (GPCR) family. Two receptor subtypes have been identified: orexin receptor type 1 (OX1R) and orexin receptor type 2 (OX2R) [25,26]. Both receptors possess the characteristic seven-transmembrane (7TM) architecture of class A GPCRs, comprising an extracellular N-terminus, seven transmembrane α-helices, three extracellular loops (ECLs), three intracellular loops (ICLs), and an intracellular C-terminal domain. The extracellular N-terminus, ECLs, and transmembrane domains primarily mediate ligand recognition and binding, whereas the intracellular loops and C-terminus are responsible for G-protein coupling and downstream signal transduction (Figure 2). The crystal structure of human OX2R in complex with suvorexant provided direct structural evidence for the organization of the orthosteric binding pocket and the binding mode of a clinically used DORA [27].

#### 2.1.2. Functional Characteristics of Orexin Receptors

OX1R is encoded by the HCRTR1 gene and is predominantly expressed in brain regions associated with emotion, reward, motivation, and stress responses, including the locus coeruleus, ventral tegmental area, prefrontal cortex, and limbic system [25,28,29]. Activation of OX1R enhances neuronal excitability and contributes to the regulation of arousal, emotional processing, reward-related behaviors, and adaptive responses to stress. Consequently, OX1R primarily modulates emotion-, stress-, and reward-associated arousal rather than serving as the principal receptor responsible for maintaining sustained wakefulness [25,28,29].

In contrast, OX2R is encoded by the HCRTR2 gene and is highly expressed in wake-promoting nuclei, including the tuberomammillary nucleus, dorsal raphe nucleus, locus coeruleus, as well as other hypothalamic and brainstem regions involved in arousal regulation [25,30]. OX2R plays a central role in maintaining sustained wakefulness, stabilizing sleep–wake state transitions, and preventing inappropriate transitions from wakefulness to sleep. Loss or dysfunction of OX2R signaling results in fragmented wakefulness and narcolepsy-like phenotypes, highlighting its indispensable role in preserving sleep–wake homeostasis [31,32].

Upon activation, orexin receptors couple to multiple G proteins in a cell type- and context-dependent manner, among which the Gq/11–PLC signaling pathway is one of the best-characterized intracellular signaling cascades [25,26]. Activation of Gq/11 stimulates phospholipase C (PLC), leading to the production of inositol trisphosphate (IP_3_) and diacylglycerol (DAG), followed by intracellular Ca^2+^ mobilization and protein kinase C (PKC) activation. In addition, both OX1R and OX2R regulate several other downstream signaling pathways, including MAPK/ERK, PI3K/Akt, and cAMP-dependent signaling [25,26]. Collectively, these signaling cascades enhance neuronal excitability, facilitate neurotransmitter release, and sustain the activity of wake-promoting neural circuits.

From a methodological perspective, label-free dynamic mass redistribution assays have been successfully applied to systematically characterize the signaling profiles of other class A GPCR antagonists, such as angiotensin AT1 receptor antagonists [33]. Similar integrated functional assays may therefore facilitate comparative evaluation of orexin receptor antagonists beyond conventional binding-affinity measurements.

**Figure 3:**
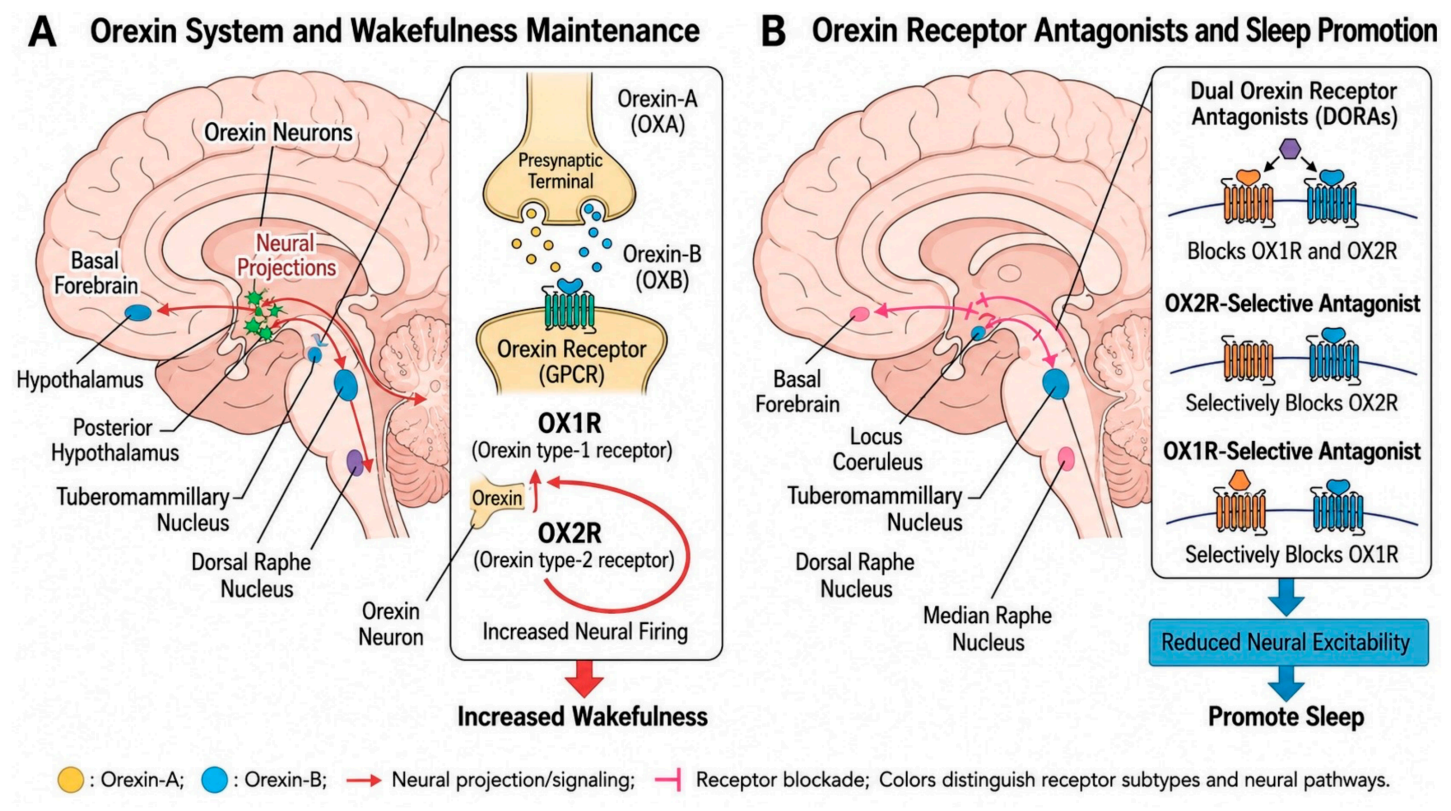
Functional organization of the orexin system and mechanisms of orexin receptor antagonists in sleep regulation. (**A**) Orexin neurons project to multiple arousal-promoting nuclei. Orexin-A and orexin-B activate OX1R and OX2R to increase neuronal excitability and maintain wakefulness. (**B**) Dual orexin receptor antagonists (DORAs), OX2R-selective antagonists, and OX1R-selective antagonists reduce neuronal excitability and promote sleep. The schematic was created by the authors based on published literature.

Although OX1R and OX2R exhibit highly conserved overall architectures, subtle differences within their ligand-binding pockets and key amino acid residues underlie their distinct ligand selectivity and functional specialization. Activation of either receptor increases neuronal activity in wake-promoting nuclei, including the locus coeruleus, dorsal raphe nucleus, and tuberomammillary nucleus, thereby strengthening arousal signaling and maintaining wakefulness (Figure 3) [25,26,30]. Functionally, OX2R primarily regulates sustained wakefulness and sleep–wake stability, whereas OX1R is more closely associated with emotional, stress-related, and reward-dependent aspects of arousal [25,28,29,31,32].

#### 2.1.3. Orexin Receptors and Insomnia

Aberrant enhancement of orexin receptor signaling has been implicated in the pathophysiology of insomnia. Persistent or excessive activation of OX2R enhances the activity of wake-promoting neural circuits, making the transition from wakefulness to sleep more difficult and thereby increasing the risk of sleep-onset insomnia, nocturnal awakenings, and impaired sleep maintenance [25,31,32]. In parallel, OX1R-mediated emotional and stress-related arousal may exacerbate insomnia associated with anxiety, psychological stress, and hyperarousal [25,28,29].

Accordingly, OX2R is considered the principal receptor responsible for the sustained hyperarousal state characteristic of insomnia, whereas OX1R appears to play a more prominent role in insomnia driven by emotional and stress-related factors. This functional distinction provides an important pharmacological rationale for the development of selective orexin receptor antagonists and dual orexin receptor antagonists as targeted therapies for insomnia.

### 2.2. Endogenous Orexin Receptor Agonists

Orexin receptor agonists are ligands capable of binding to OX1R and/or OX2R and activating their downstream signaling pathways. Within the human orexin system, Orexin-A and Orexin-B are the two endogenous agonists produced and released by orexin neurons and represent the principal neuropeptides responsible for orexin signaling [22]. By activating orexin receptors, these endogenous peptides increase the excitability of wake-promoting neurons and participate in the regulation of sleep–wake states, energy homeostasis, stress responses, reward-related behaviors, and autonomic nervous system function [25,28,30,36,37].

#### 2.2.1. Structural Characteristics of Orexin Peptides

**Figure 4:**
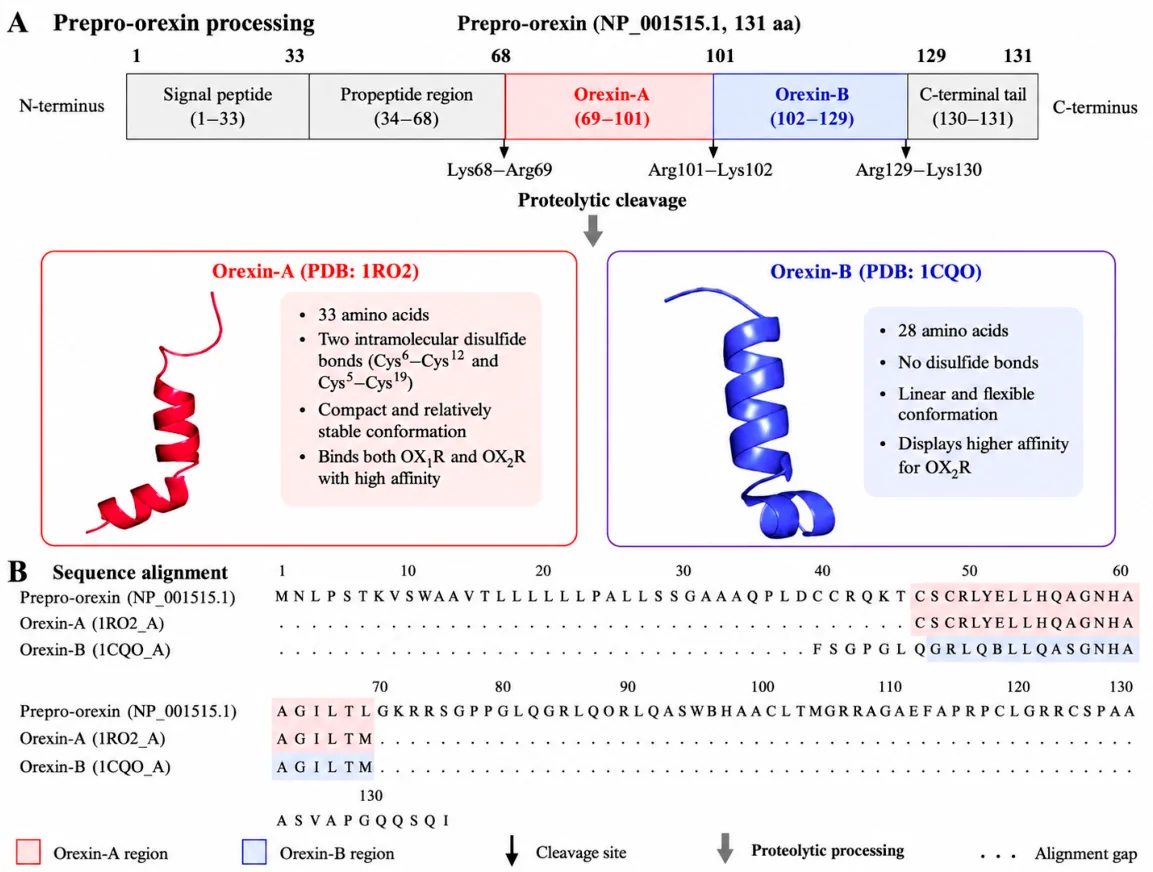
Structural characteristics and processing of human orexin peptides. (**A**) Proteolytic processing of prepro-orexin generates mature Orexin-A and Orexin-B, with their distinct structural features. (**B**) Sequence alignment of prepro-orexin, Orexin-A, and Orexin-B showing conserved regions and sequence differences. Structural models were prepared based on NMR structures reported by Kim et al. [39] and Lee et al. [40].

Both Orexin-A and Orexin-B are derived from a common precursor protein, prepro-orexin. Human prepro-orexin consists of 131 amino acids and contains the sequences of both mature neuropeptides [22,38]. Following removal of the signal peptide and subsequent proteolytic processing, prepro-orexin is cleaved to generate mature Orexin-A and Orexin-B (Figure 4) [22,38].

Orexin-A is a 33-amino-acid endogenous agonist of orexin receptors [22]. It contains four highly conserved cysteine residues that form two intramolecular disulfide bonds (Cys6–Cys12 and Cys7–Cys14) (Figure 4) [22,39]. In addition, Orexin-A undergoes N-terminal pyroglutamyl modification and C-terminal amidation. These structural features restrict the conformational flexibility of the peptide, giving rise to a compact and conformationally stable structure that is essential for efficient receptor recognition and biological activity [22,39].

Orexin-B is another endogenous orexin receptor agonist consisting of 28 amino acids [22]. Unlike Orexin-A, Orexin-B lacks intramolecular disulfide bonds. Rather than adopting a fully extended linear conformation, its solution structure is composed predominantly of two α-helices connected by a short linker region (Figure 4) [40]. Orexin-B also undergoes C-terminal amidation, which is important for maintaining receptor recognition and biological activity [22,40]. The characteristic two-helix conformation of Orexin-B has been confirmed by two-dimensional nuclear magnetic resonance (2D NMR) studies [40].

#### 2.2.2. Functional Characteristics of Orexin Peptides

Orexin-A promotes wakefulness and participates in the regulation of feeding behavior, energy homeostasis, stress responses, reward-related behaviors, and autonomic nervous system activity [25,28,29,30,36,37,41]. It efficiently activates both OX1R and OX2R, thereby contributing to OX1R-mediated regulation of emotion, reward, and stress, as well as OX2R-mediated maintenance of sustained wakefulness and sleep–wake stability [22,25,26].

Like Orexin-A, Orexin-B exerts a potent wake-promoting effect but is more closely associated with the maintenance of sustained wakefulness and stabilization of sleep–wake transitions [25,26,30]. With respect to receptor activation, Orexin-A efficiently activates both OX1R and OX2R, whereas Orexin-B displays agonistic activity toward OX2R comparable to that of Orexin-A but substantially weaker activity at OX1R, as illustrated in Figure 3A [22,26]. Consequently, Orexin-B exhibits a clear functional preference for OX2R, and its physiological actions are primarily mediated through OX2R-dependent wakefulness-maintaining pathways.

## 3. Orexin Receptor Antagonists

Orexin receptor antagonists are compounds that bind to orexin receptors and inhibit receptor activation by the endogenous ligands Orexin-A and Orexin-B, thereby suppressing orexin-mediated wake-promoting signaling [18,26,42].

Unlike conventional sedative–hypnotic agents, which produce widespread central nervous system suppression primarily by enhancing GABAergic inhibitory signaling, ORAs regulate sleep–wake homeostasis by selectively targeting the orexinergic system within the lateral hypothalamus [43]. This mechanism provides a more physiologically specific therapeutic strategy [17,44,45]. Because ORAs suppress orexin-mediated wake-promoting signaling rather than broadly reducing central nervous system excitability, they generally exert less influence on cognitive function and motor coordination and may be associated with lower residual effects and abuse potential [20,44,46].

**Figure 5:**
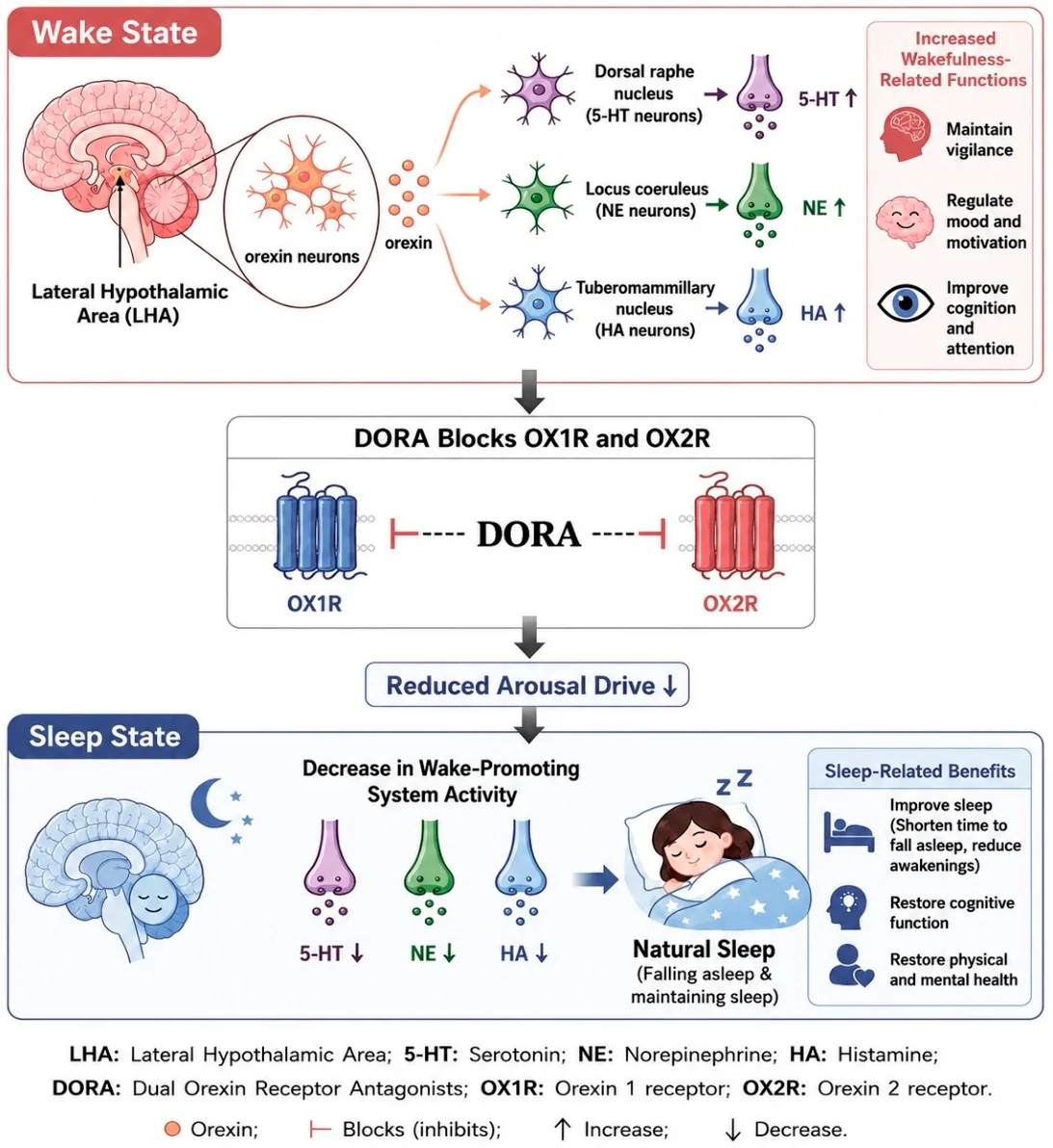
Schematic illustration of the mechanisms by which DORAs promote sleep through suppression of wake-promoting drive. The schematic was created by the authors based on published literature.

Accordingly, ORAs can be classified into three categories. The first category comprises DORAs, which simultaneously inhibit OX1R and OX2R and currently represent the major class used in clinical practice (Figure 5). The second category includes selective orexin receptor 2 antagonists (2-SORAs), which primarily target the core pathways responsible for wakefulness maintenance. Genetic evidence has demonstrated that loss-of-function mutations in OX2R can induce narcolepsy-like phenotypes, indicating that this receptor subtype serves as a critical target in sleep–wake regulation [47]. The third category consists of selective orexin receptor 1 antagonists (1-SORAs), which remain under investigation and are mainly involved in the regulation of stress responses, emotional processes, and addiction-related behaviors [48]. These agents exhibit potential therapeutic value in substance dependence and anxiety disorders but have not yet reached clinical application [49].

Following clarification of their mechanisms of action, ORAs have gradually progressed from experimental research to clinical application, forming a developmental landscape in which approved therapeutics and investigational candidates coexist.

### 3.1. Approved Drugs

Currently approved ORAs mainly consist of DORAs, including suvorexant, lemborexant, and daridorexant.

From the perspective of structural pharmacology, these compounds are predominantly lipophilic non-peptide small molecules whose molecular designs exhibit highly conserved structure–activity relationship (SAR) characteristics. Despite differences in their core scaffolds, these molecules generally adopt compact “U-shaped” or “horseshoe-shaped” conformations upon binding to OX1R/OX2R [50,51], facilitating insertion into the hydrophobic binding pockets of G protein-coupled receptors and constituting a key structural basis for their antagonistic activity. Typical pharmacophores consist of two aromatic rings connected by a central scaffold, in which π-π stacking interactions between aromatic moieties contribute to conformational stabilization and enhanced receptor complementarity, while polar groups improve binding selectivity and affinity through hydrogen bonding or electrostatic interactions [50,51].

**Figure 6:**
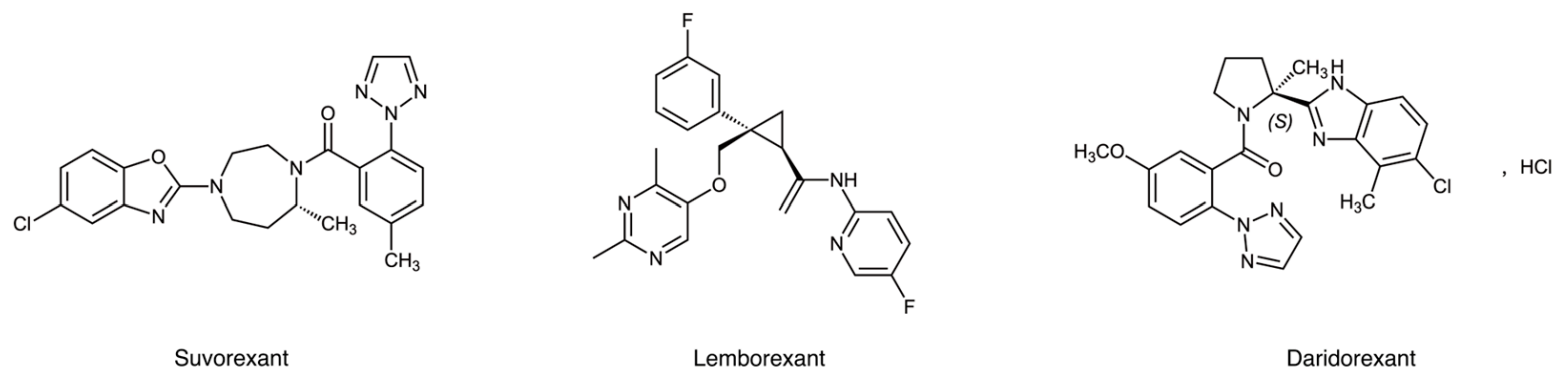
Chemical structures of the clinically approved orexin receptor antagonists suvorexant, lemborexant, and daridorexant.

Suvorexant (Figure 6) was the first approved dual orexin receptor antagonist and exhibits comparable antagonistic activity toward OX1R and OX2R, with Ki values of approximately 1.1 nM and 1.4 nM, respectively [52], indicating minimal subtype selectivity [52]. The clinically recommended dosage ranges from 10 to 20 mg [20]. Suvorexant is primarily metabolized by CYP3A4 [20] and possesses a relatively long elimination half-life (approximately 12–15 h [10]). Although this pharmacokinetic characteristic ensures sustained nocturnal efficacy, it may also contribute to residual next-day effects in some patients [20].

Mechanistically, suvorexant simultaneously antagonizes OX1R and OX2R by competitively blocking the binding of Orexin-A and Orexin-B to their receptors, thereby suppressing orexin-mediated wake-promoting signaling pathways and reducing central wakefulness drive [18,42]. Preclinical and clinical studies have demonstrated that suvorexant significantly shortens latency to persistent sleep (LPS), reduces wake after sleep onset (WASO), and increases total sleep time (TST), exhibiting stable efficacy in improving sleep initiation difficulties [53].

Structurally, the hydrophobic aromatic scaffold contributes to receptor binding affinity and forms an important structural basis for DORAs [51], thereby enhancing receptor affinity and blood–brain barrier permeability and establishing the fundamental design paradigm for DORA compounds [54]. The central seven-membered 1,4-diazepane ring functions as a critical linker and plays an important role in maintaining molecular conformational stability. Based on this scaffold, medicinal chemists have continuously optimized molecular structures through strategies such as “scaffold hopping” [55]. For example, within 3-aryl pyrazolidine scaffolds, 4′-methoxy substitution on aromatic rings is essential for maintaining antagonistic activity, whereas substitution at the pyrazolidine N-1 position significantly influences metabolic stability and is closely associated with CYP3A4 inhibitory activity [55].

Overall, suvorexant has demonstrated well-established clinical efficacy in the treatment of insomnia. However, its relatively long elimination half-life necessitates careful consideration of potential next-day residual effects in clinical practice. Recent advances in high-resolution cryo-electron microscopy (cryo-EM) have further elucidated the molecular basis of suvorexant binding to orexin receptors. Structural studies have revealed that suvorexant occupies a deep hydrophobic pocket within the transmembrane domain of OX2R and adopts a characteristic horseshoe-like conformation within the receptor cavity [34]. This binding mode enables stable occupation of the orthosteric binding site and facilitates extensive hydrophobic interactions and hydrogen-bonding networks with key residues located in the TM3, TM5, and TM6 helices, thereby preventing the conformational rearrangements required for receptor activation [34,35]. The deep-binding configuration provides a structural explanation for the high affinity of suvorexant toward both OX1R and OX2R and may also contribute to its prolonged pharmacological activity and sustained hypnotic effects [34,51]. Consequently, while the strong receptor occupancy of suvorexant is advantageous for maintaining sleep throughout the night, it may also increase the likelihood of next-day residual sedation and related adverse effects [10,34].

Lemborexant (Figure 6) exhibits higher affinity toward OX2R and therefore demonstrates a degree of subtype selectivity [51]. The clinically recommended dosage ranges from 5 to 10 mg [56]. Lemborexant is primarily metabolized by CYP3A4 [20] and has a half-life of approximately 17–19 h [10]. Clinical studies indicate that lemborexant is superior to suvorexant in improving sleep maintenance, particularly in patients with early morning awakening insomnia.

From a structural design perspective, structural optimization of lemborexant contributes to enhanced receptor-binding stability and distinct binding kinetics [50,51]. In addition, lemborexant exhibits rapid and reversible binding kinetics, which may contribute to its lower residual next-day effects [51]. Similar to suvorexant, lemborexant simultaneously antagonizes OX1R and OX2R through competitive inhibition of Orexin-A and Orexin-B binding, thereby suppressing orexin-mediated wake-promoting signaling pathways and reducing central wakefulness drive [18].

Notably, preferential binding toward OX2R enables more effective modulation of wakefulness maintenance, thereby conferring a certain degree of “functional selectivity” at the mechanistic level [51]. Functional selectivity (also referred to as biased signaling) describes the ability of different ligands to stabilize distinct receptor conformations, thereby preferentially activating specific downstream signaling pathways. Consequently, selective OX2R antagonists are expected to maintain sleep-promoting efficacy while minimizing adverse effects associated with OX1R inhibition.

Regarding pharmacodynamic and clinical efficacy, lemborexant significantly improves multiple sleep parameters. Studies have shown that it reduces LPS, decreases WASO, and increases TST, with particularly pronounced efficacy in improving sleep maintenance [56]. Compared with suvorexant, lemborexant exhibits advantages in prolonging sleep duration and reducing nocturnal awakenings, particularly in patients with predominant early awakening symptoms. Furthermore, its limited influence on REMS contributes to preservation of a sleep architecture closer to physiological conditions [42].

Compared with suvorexant, structural studies of lemborexant have provided further insights into the molecular determinants underlying its preferential interaction with OX2R. The cryo-EM structure of the lemborexant-OX2R complex, resolved in 2022, demonstrated that lemborexant also occupies the deep hydrophobic binding pocket within the receptor transmembrane domain; however, notable differences were observed in the length and flexibility of its linker region, aromatic ring substitution patterns, and the distribution of hydrogen-bond donor and acceptor groups [35]. These structural characteristics influence ligand dissociation kinetics and receptor occupancy profiles, thereby contributing to enhanced OX2R selectivity and potentially reducing the risk of next-day residual effects [35]. Furthermore, the relatively rapid and reversible binding kinetics of lemborexant may help maintain sleep continuity while minimizing impairment of daytime cognitive and psychomotor performance [35,56]. Consequently, the favorable efficacy of lemborexant in improving sleep maintenance appears to be associated not only with its pharmacokinetic properties but also with the optimization of its receptor-binding interactions and occupancy dynamics [35,56].

Overall, DORAs improve sleep by selectively suppressing orexin-mediated arousal signaling without inducing the widespread central nervous system inhibition typically associated with conventional sedative–hypnotic agents. Moreover, differences in receptor-binding modes, binding kinetics, and structure–activity relationships contribute substantially to the distinct efficacy and safety profiles observed among individual DORAs [35].

Daridorexant (Figure 6) is characterized as a short half-life DORA, with an elimination half-life of approximately 8 h [10]. Reduced systemic exposure contributes to decreased drug accumulation and lower residual next-day effects [18,42], while clinical studies have additionally demonstrated improvements in daytime functioning. Daridorexant simultaneously antagonizes OX1R and OX2R and exhibits partial OX2R preference. The recommended clinical dosage ranges from 25 to 50 mg [57], and the compound is predominantly metabolized by CYP3A4 [20], thereby reducing systemic accumulation and minimizing adverse events such as next-day somnolence.

Structurally, daridorexant achieves a balance between receptor-binding affinity and pharmacokinetic properties through optimization of molecular polarity and metabolic characteristics, contributing to improved pharmacokinetic performance and reduced residual next-day effects [58].

Mechanistically, daridorexant competitively inhibits binding of Orexin-A and Orexin-B to OX1R/OX2R, thereby suppressing orexin-mediated wake-promoting signaling and reducing central wakefulness levels [18,42]. Clinical studies have demonstrated that daridorexant significantly reduces LPS and WASO while increasing TST, and additionally provides benefits in improving daytime functioning, which may be associated with its lower residual next-day effects [57].

Overall, daridorexant represents a notable advancement within the DORA class, achieving effective insomnia treatment while enhancing the safety profile through pharmacokinetic optimization. Compared with suvorexant and lemborexant, daridorexant exemplifies the evolution of DORA drug design from a primary focus on maximizing receptor affinity toward a more balanced strategy that integrates pharmacokinetic optimization and clinical tolerability. Through modulation of molecular polarity, reduction in lipophilicity, and refinement of hydrogen-bonding interactions, daridorexant maintains potent antagonistic activity at both OX1R and OX2R while exhibiting a shorter elimination half-life [58]. This reduced systemic exposure limits residual plasma drug concentrations upon awakening, thereby decreasing the risk of daytime somnolence, cognitive impairment, and other next-day residual effects [57,59,60]. The development of daridorexant highlights a broader trend in the design of next-generation DORAs, in which the therapeutic objective has shifted from simply enhancing hypnotic efficacy to achieving an optimal balance between nocturnal sleep improvement and preservation of daytime functioning [29,58,61].

At the mechanistic level, DORAs competitively antagonize OX1R and OX2R, inhibit downstream G protein-mediated signaling pathways, and reduce excitability of wake-promoting neurons, resulting in hyperpolarization and decreased firing frequency [62]. This process predominantly occurs within the “wakefulness switch” pathways formed by orexin neurons located in the lateral hypothalamus [26]. Owing to their pathway-specific mechanisms rather than global central nervous system suppression, DORAs exhibit significantly reduced adverse effects related to cognitive impairment [63], respiratory depression [64], and dependence compared with conventional GABAergic sedative–hypnotics.

In summary, ORAs achieve precise modulation of the sleep–wake system through selective inhibition of OX1R/OX2R-mediated wake-promoting signaling. Their chemical structural characteristics directly determine receptor selectivity, binding affinity, and in vivo metabolic behavior, thereby influencing clinical efficacy and safety profiles (Table 1).

### 3.2. Investigational Drugs in Clinical Trials

**Figure 7:**
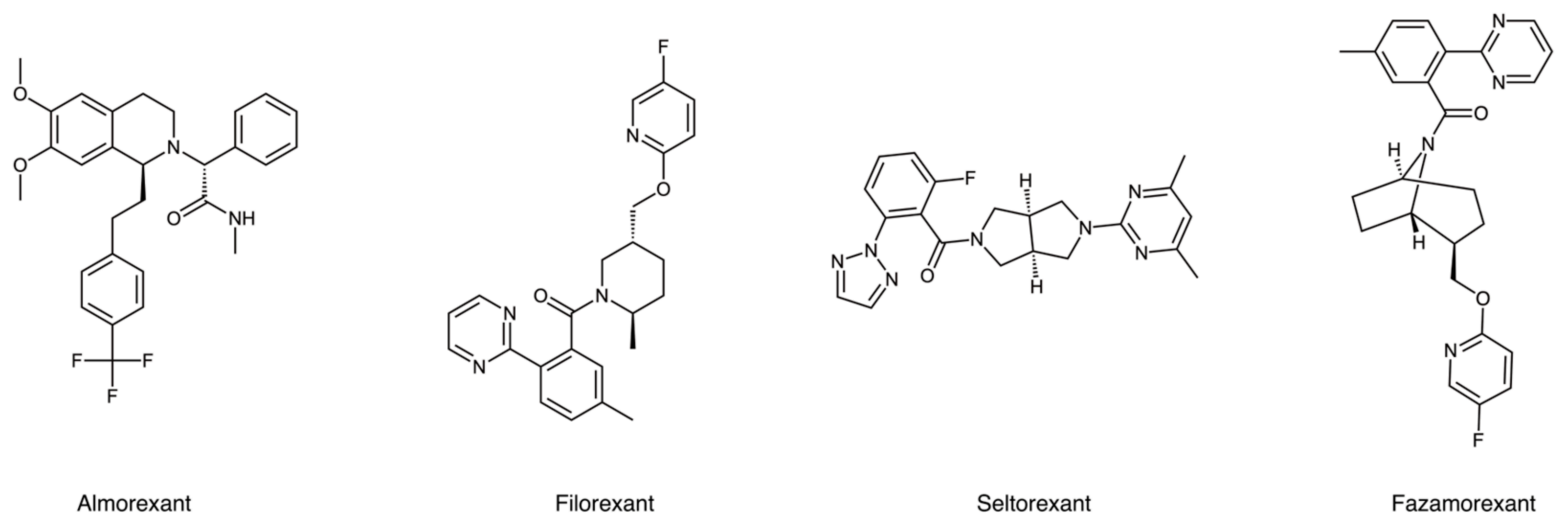
Chemical structures of representative investigational orexin receptor antagonists currently in clinical development: almorexant, filorexant, seltorexant and fazamorexant.

In addition to approved DORAs, multiple candidate compounds remain under clinical investigation and continue to drive the optimization and development of this drug class [71]. Almorexant (Figure 7), an early representative DORA compound, demonstrated favorable sleep-promoting effects in clinical studies [72]. However, long-term investigations revealed tolerability concerns, including elevated liver enzyme levels [73]. Pharmacokinetic studies showed that systemic exposure increased approximately 2.8-fold and 7.2-fold in patients with mild and moderate hepatic impairment, respectively [73], suggesting potential hepatotoxicity risk and ultimately preventing its clinical approval.

Subsequently, filorexant (Figure 7) entered mid- to late-stage clinical development and exhibited certain efficacy in improving sleep outcomes in Phase II studies [74]. However, its pharmacodynamic and pharmacokinetic characteristics were largely comparable to those of suvorexant, another Merck-developed compound, resulting in limited differentiation advantages [69,71]. Furthermore, studies evaluating filorexant in alternative indications failed to demonstrate significant therapeutic benefits, and the compound was eventually discontinued and removed from the development pipeline [74]. These findings suggest that despite the clear mechanistic advantages of DORAs, successful clinical translation remains highly dependent on achieving an optimal balance between pharmacokinetic properties and safety profiles [71,74].

From the perspective of structural pharmacology, investigational DORA compounds generally maintain the design principles established by approved agents, namely lipophilic non-peptide small-molecule scaffolds capable of adopting “U-shaped” or “horseshoe-shaped” conformations to fit the hydrophobic binding pockets of OX1R/OX2R [52]. However, next-generation candidate compounds increasingly emphasize optimization of pharmacokinetic properties, such as modulation of molecular polarity, reduction in lipophilicity, or incorporation of readily metabolizable groups to shorten half-life and reduce residual next-day effects [53,71]. In addition, some studies have attempted to improve OX2R selectivity through structural modification, thereby preserving hypnotic efficacy while minimizing interference with emotional and stress-related pathways [75].

For example, the 2-SORA seltorexant (Figure 7), developed for insomnia and major depressive disorder (MDD) accompanied by insomnia symptoms, represents an important development direction in this field [75,76]. Seltorexant is capable of crossing the blood–brain barrier and binds OX2R with high affinity, suppressing excessive arousal states through inhibition of OX2R-mediated wake-promoting signaling, thereby reducing LPS and prolonging sleep maintenance [75].

Randomized controlled clinical trials demonstrated that both 10 mg and 20 mg doses of seltorexant significantly improved LPS and WASO in patients with insomnia, with efficacy superior to placebo and sustained over two weeks [77]. Common adverse effects include mild headache and fatigue, while overall tolerability appears favorable [77]. In Phase III clinical studies, combination therapy with antidepressants significantly improved both depressive symptoms and sleep disturbances and was associated with a relatively low incidence of adverse events [75].

Building upon these advances, the development of orexin receptor antagonists by Chinese pharmaceutical companies has increasingly shifted from conventional “me-too” strategies toward rational drug design guided by SAR optimization. Among the emerging candidates, KHN707, developed by Kanghong Pharmaceutical, represents a notable selective OX2R antagonist [78,79,80]. At the time of the literature search conducted for this review, the detailed chemical structure of KHN707 had not been publicly disclosed by the developer or reported in accessible published sources; therefore, KHN707 is discussed in the text but is not included in Figure 7. As a Class 1 innovative chemical entity in China, KHN707 has been designed to promote sleep through selective inhibition of OX2R while minimizing interference with OX1R-mediated signaling, thereby potentially reducing the risk of adverse events such as next-day somnolence and residual sedation [81,82]. Unlike conventional DORAs, which simultaneously antagonize both OX1R and OX2R, KHN707 emphasizes enhanced functional occupancy of OX2R, directly targeting the core neural circuitry governing sleep–wake regulation [79,83]. Accumulating evidence suggests that OX2R plays a predominant role in sleep induction and maintenance, whereas OX1R is more closely associated with reward processing, emotional regulation, and stress responses. Consequently, improving OX2R selectivity has emerged as a key objective in the development of next-generation ORAs and may enable the preservation of hypnotic efficacy while minimizing unintended modulation of reward- and emotion-related pathways [83,84]. In addition, KHN707 incorporates a heterocyclic scaffold optimized for improved metabolic stability, which may reduce residual plasma drug concentrations upon awakening and lower the likelihood of hangover-like effects, thereby enhancing overall tolerability without compromising therapeutic efficacy [78,79]. Nevertheless, KHN707 remains in the early stages of clinical development, and comprehensive data regarding its efficacy and long-term safety in humans are still limited.

In parallel, the clinical translation of domestically developed DORAs has progressed rapidly. Fazamorexant (Figure 7), a next-generation short-acting DORA, has demonstrated promising efficacy and a favorable residual-effect profile in late-stage clinical development [85]. As a dual antagonist targeting both OX1R and OX2R, fazamorexant incorporates a flexible heteroaromatic framework and optimized central nervous system exposure, enabling effective blockade of Orexin-A- and Orexin-B-mediated receptor activation while reducing systemic drug accumulation through a shortened elimination half-life [79,86]. By suppressing excessive orexin-driven arousal signaling, fazamorexant facilitates sleep initiation and maintenance [71]. In a multicenter, randomized, double-blind, placebo-controlled Phase III trial involving 1034 adult patients with insomnia, treatment with fazamorexant reduced sleep onset latency by approximately 46% and decreased nocturnal awakenings by approximately 42%, with sustained efficacy observed during long-term administration [86]. Furthermore, its relatively rapid metabolism and shorter half-life may contribute to a lower incidence of next-day residual effects and daytime impairment [71]. In contrast to earlier-generation DORAs, which primarily focused on prolonging total sleep time, fazamorexant places greater emphasis on minimizing morning residual effects and preserving daytime cognitive performance. This design philosophy reflects the ongoing evolution of domestically developed ORAs from a predominantly hypnotic efficacy-oriented approach toward a more comprehensive strategy aimed at improving overall sleep quality and maintaining next-day functioning [78,87]. At present, fazamorexant has completed its new drug application process and is under regulatory review.

Overall, investigational ORAs in clinical development are progressively evolving toward higher selectivity, shorter half-lives, and lower residual effects, while maintaining the classical structural framework of DORAs [71,75]. Among these, selective OX2R antagonists emphasize safety optimization, whereas optimized DORA compounds focus on improving pharmacokinetic properties while preserving therapeutic efficacy. These strategies provide important directions for the development of next-generation anti-insomnia therapeutics (Table 2).

## 4. Clinical Applicability of Orexin Receptor Antagonists

As a novel class of therapeutics for insomnia, ORAs exhibit substantial advantages over conventional sedative–hypnotic agents in terms of overall safety profiles and applicability across patient populations. However, caution remains necessary in older adults and patients with hepatic impairment. Because these agents are primarily metabolized by CYP3A4, systemic drug exposure may increase in such populations, necessitating initiation at lower doses followed by individualized dose adjustment [20].

First, regarding insomnia phenotypes, ORAs demonstrate more pronounced therapeutic benefits in patients predominantly experiencing sleep maintenance insomnia. By contrast, their advantages in individuals with isolated sleep-onset insomnia appear relatively limited; therefore, treatment selection should be tailored according to specific symptom profiles. Furthermore, ORAs provide important alternative therapeutic options for patients who are intolerant to conventional hypnotics or exhibit elevated treatment-related risks. Because ORAs do not target the GABA_A_ receptor complex, they are less likely to induce pronounced muscle relaxation, ataxia [34], or cognitive impairment [44,63]. Consequently, they are particularly suitable for older adults [78,79], patients exhibiting tolerance or dependence risk associated with benzodiazepines or Z-drugs [20,46], and individuals with chronic insomnia requiring long-term treatment while aiming to minimize dependence risk [60,78].

Additionally, ORAs exhibit relative safety advantages in patients with concomitant respiratory disorders or increased susceptibility to central nervous system depression. Since the orexin system is not directly involved in basal respiratory regulation, these agents are associated with a lower incidence of respiratory depression [44]. Therefore, compared with conventional sedative–hypnotics, ORAs may offer improved safety profiles in patients with obstructive sleep apnea (OSA) or chronic obstructive pulmonary disease (COPD) [44,75]. Nevertheless, individualized assessment based on disease severity remains essential.

Moreover, among patients requiring preservation of daytime functioning, ORAs characterized by shorter half-lives or optimized pharmacokinetic properties may offer additional advantages, as reduced residual next-day effects contribute to lower incidences of daytime somnolence and impaired attention [57,70].

Overall, the primary target populations for ORA therapy include patients with insomnia predominantly characterized by sleep maintenance impairment, individuals intolerant to GABAergic agents or at risk of dependence, and special populations requiring enhanced medication safety, such as older adults and patients with concomitant respiratory disorders.

## 5. Future Directions and Challenges in Orexin Receptor Antagonist Development

Although ORAs have demonstrated favorable clinical efficacy and safety in insomnia treatment, currently available DORAs primarily suppress wake-promoting drive through simultaneous inhibition of OX1R and OX2R. Limitations remain regarding the fine regulation of sleep architecture [88], interindividual variability in treatment response [89], and long-term therapeutic efficacy. In addition, some patients continue to experience residual next-day effects and dose-dependent sedative responses, suggesting that the functional selectivity of current receptor-blocking strategies still has room for improvement.

Therefore, future research should focus on the orexin receptor system itself, with further optimization of receptor selectivity and drug characteristics.

### 5.1. Highly Selective Orexin Receptor Antagonists

With increasing understanding of the mechanisms through which the orexin system regulates sleep–wake homeostasis, precision drug design based on receptor functional divergence has gradually emerged as an important direction in anti-insomnia drug development. The orexin system exerts its physiological functions primarily through two G protein-coupled receptors, namely OX1R and OX2R. Among them, OX2R plays a dominant role in maintaining wakefulness and serves as the core receptor regulating sleep–wake homeostasis, whereas OX1R is more closely associated with emotional regulation, stress responses, and reward-related neural pathways [71,75]. This distinct functional division provides an important theoretical basis for targeted drug design, suggesting that preferential modulation of OX2R alone may achieve major therapeutic benefits in sleep induction, whereas intervention at OX1R may not be essential [31].

Currently approved DORAs, including suvorexant, lemborexant, and daridorexant, effectively suppress orexin-mediated wake-promoting signaling through simultaneous inhibition of OX1R and OX2R, thereby improving both sleep initiation and sleep maintenance disorders and demonstrating favorable efficacy and tolerability in clinical settings [54,56]. However, this “dual receptor blockade” strategy fundamentally represents a relatively non-selective form of neural regulation. While reducing wake-promoting drive, it may also influence emotional and stress-related pathways mediated by OX1R, thereby limiting opportunities for further optimization of safety and functional specificity [31,71]. Moreover, some patients continue to experience dose-dependent adverse effects, including residual next-day somnolence and reduced attention, suggesting that current therapeutic approaches still require improvement regarding target precision [66,70].

Based on these mechanistic insights and clinical limitations, 2-SORAs have gradually become a major focus of research. By preferentially or selectively inhibiting OX2R-mediated wake-promoting signaling pathways, these compounds may theoretically preserve hypnotic efficacy while minimizing interference with OX1R-related neural networks, thereby enabling more precise modulation of the sleep–wake system [31,75]. Fundamentally, this strategy represents a transition from “broad suppression of wakefulness” toward “targeted regulation of core wake-promoting nodes”, reflecting the broader evolution of neuropsychiatric drug design from generalized intervention to precision modulation.

From a pharmacological perspective, highly selective OX2R antagonists may offer several potential advantages. First, regarding efficacy, because OX2R serves as the key receptor maintaining wakefulness, selective inhibition of this receptor alone may substantially reduce wake-promoting drive, thereby shortening LPS, reducing WASO, and increasing TST, particularly with respect to improving sleep maintenance [32,54,77].

Second, in terms of safety, avoiding inhibition of OX1R may reduce potential interference with emotional regulation, reward pathways, and stress responses, thereby lowering the risk of anxiety fluctuations, mood disturbances, and related neuropsychiatric adverse effects [32,71].

Third, selective targeting of wake-promoting pathways rather than broad suppression of central nervous system activity may contribute to preservation of sleep architecture closer to physiological conditions, particularly through reduced interference with REM sleep. This feature may be important for emotional stability and memory consolidation [32,42].

Furthermore, from a clinical perspective, highly selective antagonists may also provide advantages in preserving daytime functioning. Reduced interaction with non-target receptors could theoretically decrease nonspecific inhibitory effects within the central nervous system, thereby minimizing residual next-day somnolence, cognitive impairment, and deficits in motor coordination [6,32,66,70]. This characteristic is especially important for patients who require maintenance of daytime alertness. At the same time, selective targeting may reduce treatment variability arising from differences in receptor expression among individuals, thereby improving predictability of therapeutic responses.

Representative selective OX2R antagonists such as seltorexant have already demonstrated favorable efficacy and safety in clinical studies, significantly improving both sleep initiation and sleep maintenance parameters while exhibiting good overall tolerability without obvious severe central nervous system depressant-related adverse events [77,90]. These findings provide clinical validation for selective intervention strategies based on receptor functional divergence and further support the future therapeutic potential of this approach in insomnia treatment.

Overall, highly selective OX2R antagonists have become a major research focus. Compared with conventional DORAs, they may further optimize safety and functional selectivity while maintaining efficacy and represent an important trend in insomnia therapy, transitioning from “broad-spectrum blockade” toward “precision modulation” [31,90].

### 5.2. Pharmacokinetic Optimization

The clinical efficacy and tolerability of orexin receptor antagonists are strongly influenced by their pharmacokinetic properties, including oral absorption, blood–brain barrier penetration, central nervous system distribution, metabolism, and elimination [46,51,58]. Because orexin receptors are predominantly located in the brain, sufficient blood–brain barrier permeability is essential for achieving effective receptor occupancy. However, excessive lipophilicity or prolonged central exposure may increase nonspecific tissue distribution and extend receptor inhibition beyond the intended sleep period. Therefore, optimal drug design should balance rapid brain entry with controlled central exposure and timely clearance before awakening [51,58].

Approved orexin receptor antagonists are orally active, lipophilic small molecules that are predominantly metabolized through CYP3A4-related pathways [46]. Their pharmacokinetic profiles may therefore be influenced by food intake, hepatic function, and concomitant administration of CYP3A4 inhibitors or inducers. High plasma protein binding and interindividual differences in metabolic capacity may further contribute to variability in systemic and central drug exposure [46,69]. These factors should be considered when selecting doses for older adults, patients with hepatic impairment, and individuals receiving multiple medications.

Future pharmacokinetic optimization should focus on achieving rapid absorption after bedtime, sufficient but not excessive blood–brain barrier penetration, limited formation of centrally active metabolites, predictable receptor occupancy, and efficient clearance before the next waking period [58,69]. Importantly, residual next-day effects are not determined by elimination half-life alone, but also by unbound drug concentrations, brain distribution, receptor-binding kinetics, and the persistence of active metabolites [46,51]. Integrated pharmacokinetic–pharmacodynamic modeling and brain receptor-occupancy studies may therefore facilitate the development of next-generation compounds that maintain nocturnal efficacy while minimizing daytime impairment [58].

### 5.3. Safety and Long-Term Clinical Considerations

From the perspective of medication safety, the safety profiles of ORAs may vary among different patient populations. Older adults generally exhibit reduced drug metabolism capacity and increased sensitivity to sedative effects; therefore, lower initial dosages are commonly recommended in clinical practice, followed by gradual adjustment according to tolerability [83]. When ORAs are co-administered with other central nervous system depressants, potential additive effects should be considered to avoid excessive sedation. Furthermore, because these agents are primarily metabolized through hepatic enzyme systems, careful evaluation of drug–drug interactions involving CYP3A4-related medications is necessary due to potential effects on systemic drug concentrations [20,91].

Current clinical evidence indicates that the overall incidence of adverse events associated with suvorexant, lemborexant, and daridorexant at standard therapeutic doses is generally comparable to placebo, with commonly reported adverse effects including mild headache, somnolence, and dry mouth [46]. Nevertheless, residual next-day somnolence may still occur and exhibits dose dependency, becoming more pronounced at higher doses or when combined with other central nervous system depressants [53,56]. These findings suggest that the impact of ORAs on daytime functioning requires individualized assessment and dosage optimization.

Although ORAs generally exhibit low abuse potential [60,78], and subjective euphoric effects associated with lemborexant are significantly lower than those observed with zolpidem—supporting routine prescription management in clinical settings [11]—daytime somnolence and impaired attention may still occur in certain individuals in a dose-dependent manner [86]. In addition, concomitant use with other central nervous system depressants, including alcohol, may significantly increase the risk of excessive sedation [66]. Therefore, co-administration should either be avoided or accompanied by rigorous risk assessment.

Notably, important evidence gaps remain regarding the long-term safety of ORAs. Current systematic evidence concerning sustained efficacy, tolerance development, and withdrawal-related responses remains relatively limited [82]. Although existing one-year studies have not demonstrated clear evidence of drug tolerance or typical rebound insomnia, these conclusions are primarily derived from highly selected clinical trial populations. Therefore, adaptive changes associated with long-term use and interindividual variability require further investigation [83].

In addition to uncertainties regarding long-term treatment, several uncommon but clinically relevant adverse events have been reported during post-marketing surveillance, including sleep paralysis and cataplexy-like events. Post-marketing pharmacovigilance analyses have identified sleep paralysis as a prominent safety signal associated with DORAs; however, spontaneous reporting data cannot be used to estimate its true incidence, most frequently occurring during transitions between REM sleep and wakefulness, potentially reflecting transient disruption of REM regulatory mechanisms [92]. More rarely, cataplexy-like manifestations—including sudden muscle weakness and head dropping—suggest that acute suppression of the orexin system may contribute to specific adverse effect profiles in susceptible individuals [93].

Regarding rebound insomnia after treatment discontinuation, available evidence remains limited but generally suggests a low risk of clinically significant rebound or withdrawal symptoms. Multiple Phase III clinical trials and extension studies evaluating daridorexant and suvorexant reported no evidence of typical rebound insomnia or withdrawal symptoms after discontinuation at the end of standard treatment periods [52,94]. Similarly, short-term studies suggested that lemborexant did not induce substantial deterioration of sleep parameters following treatment cessation [95].

Nevertheless, these findings should be interpreted cautiously because most studies involved short- or medium-term follow-up and excluded patients with complex comorbidities. Consequently, in real-world settings, some patients may experience transient worsening of sleep initiation following discontinuation. Such phenomena are more likely to reflect recurrence of primary insomnia rather than classical pharmacological rebound effects. In addition, during transitions from benzodiazepines or Z-drugs to ORA therapy, withdrawal symptoms associated with abrupt discontinuation of prior medications may be misinterpreted as rebound effects attributable to ORAs. This clinical scenario warrants careful differentiation [96].

From a pharmacoeconomic perspective, ORAs, as novel targeted sleep-modulating therapeutics, are associated with substantially higher costs than conventional sedative–hypnotic agents. Consequently, no consensus has yet been established regarding their cost-effectiveness in long-term treatment strategies. Future studies should comprehensively evaluate sustained efficacy, safety advantages, and healthcare economic burden to define optimal treatment strategies across different patient populations.

Beyond long-term efficacy, safety, and economic considerations, several specific clinical settings also require particular attention during ORA therapy because concomitant medications or medical procedures may increase the risk of adverse events. Patients with chronic pain frequently experience comorbid insomnia and often receive medications with central nervous system depressant effects [83]. These patients are commonly treated with gabapentinoids, such as gabapentin and pregabalin, or tricyclic antidepressants. Because these medications may produce sedation and other central nervous system effects, concomitant administration with orexin receptor antagonists may increase the risk of excessive somnolence, dizziness, impaired psychomotor performance, and falls, particularly in older adults. Although direct clinical evidence specifically evaluating these combinations remains limited, prescribing information for approved ORAs recommends caution when they are administered with other CNS depressants [66,67,68,70]. Therefore, individualized dose adjustment and close clinical monitoring should be considered in patients receiving medications for chronic pain.

Perioperative management represents another important clinical scenario requiring special consideration. Because ORAs suppress wake-promoting signaling, concomitant exposure to general anesthetics, opioids, benzodiazepines, or other perioperative sedatives may produce additive central nervous system depression and could increase the risk of excessive postoperative somnolence, impaired psychomotor function, or delayed recovery. However, direct clinical evidence regarding perioperative use of ORAs remains limited, and no universally accepted discontinuation interval has been established. Therefore, preoperative medication review and individualized management by the anesthesiology team are recommended, taking into account the specific ORA, its elimination half-life, dosage, timing of administration, and the patient’s clinical condition [66,67,68,70].

Evidence regarding interactions between ORAs and recreational or illicit substances remains limited. Concomitant use with alcohol, cannabis, opioids, or other sedative substances may theoretically produce additive central nervous system depression, resulting in excessive somnolence, impaired judgment, reduced psychomotor performance, and an increased risk of accidents. A controlled study has shown that co-administration of suvorexant with alcohol can enhance daytime sleepiness and functional impairment [87]. Although human abuse-potential studies suggest that daridorexant and lemborexant generally exhibit lower or comparable abuse-related effects relative to conventional hypnotics, caution remains necessary in individuals with a history of recreational sedative use [60,61]. In contrast, interactions with stimulant drugs such as cocaine have not been adequately characterized and may produce unpredictable neurobehavioral effects. Therefore, patients should be advised to avoid recreational or illicit substances during ORA treatment until more direct clinical evidence becomes available [66,67,68,70].

Although currently available ORAs exhibit favorable short-term safety profiles, evidence regarding their long-term efficacy and safety remains limited. Large-scale, long-term clinical studies are therefore required to evaluate sustained effects on sleep architecture, the potential development of tolerance, the durability of therapeutic efficacy, and treatment outcomes in diverse patient populations, particularly older adults [88].

Collectively, future research should focus on optimized dosing strategies, individualized treatment selection, and comprehensive long-term safety evaluation. These advances may improve the durability, safety, and clinical applicability of ORA therapy across diverse patient populations.

### 5.4. Interindividual Variability and Precision Medicine Strategies

Clinical evidence indicates considerable interindividual variability in the efficacy and tolerability of ORAs, which may be associated with differences in receptor expression, neural regulation, and drug metabolism. Therefore, future treatment strategies are expected to incorporate precision medicine approaches, including individualized dose adjustment, optimized drug selection according to clinical phenotypes, and biomarker-guided therapeutic decision-making [6].

Future precision-medicine frameworks may also benefit from considering secondary biological processes that influence central nervous system vulnerability. Studies in diverse neurological models have shown that oxidative stress, neuroinflammation, blood–brain barrier disruption, autophagic degradation of endothelial tight-junction proteins, and CRTC1-CREB-BDNF signaling can be pharmacologically modulated [97,98,99,100,101]. Although these studies were not conducted with orexin receptor antagonists, they illustrate candidate mechanistic dimensions that may be examined when evaluating comorbid neurological conditions and heterogeneous treatment responses.

## 6. Conclusions

In conclusion, ORAs represent an important advancement in the pharmacological treatment of insomnia. By selectively modulating the sleep–wake regulatory system, ORAs have shifted therapeutic strategies from traditional global central nervous system suppression toward targeted regulation of wakefulness. Compared with conventional hypnotic agents, ORAs exhibit greater mechanistic specificity and have demonstrated favorable efficacy and safety profiles in clinical practice.

Although currently approved dual orexin receptor antagonists have shown significant benefits in improving both sleep onset and sleep maintenance, several challenges remain. These include residual next-day effects in certain patients, considerable inter-individual variability in treatment response, and limited evidence regarding long-term safety and sustained efficacy. Addressing these limitations will be essential for further optimizing the clinical utility of this drug class.

Future research is expected to focus on improving receptor selectivity, optimizing pharmacokinetic properties, advancing personalized treatment strategies, and generating robust long-term clinical evidence. In addition, a deeper understanding of sleep–wake regulation and its interactions with other physiological systems may further expand the therapeutic potential of orexin-targeted interventions. Overall, ORAs represent a promising and rapidly evolving class of therapeutics, with the potential to play an increasingly important role in the management of insomnia and related disorders.

## Figures and Tables

**Table 1:** Drug-like properties and clinical characteristics of approved ORAs.

Drug	Ki (nM) and Selectivity	t_1/2_	Dose	Company and Approval Year	Indications	Suitable Population	Advantages	Adverse Effects	Clinical Efficacy
Suvorexant	OX1R ≈ 1.1; OX2R ≈ 1.4; No selectivity [52]	12–15 h [10]	10–20 mg [53]	Merck 2014	Mixed insomnia; patients intolerant to GABAergic drugs or with risk of dependence [49,55]	General insomnia population [49,55]	Stable efficacy; low dependence potential; minimal food interaction [65]	Somnolence; nightmares [66]	↓ LPS; ↓ WASO; ↑ TST [66]
Lemborexant	OX2R ≈ 0.18; OX1R ≈ 3.3; OX1R/OX2R = 18 [51]	17–19 h [10]	5–10 mg [56]	Eisai 2019	Early awakening insomnia; chronic insomnia; older adults (≥65 years); mild obstructive sleep apnea [67]	Patients with sleep maintenance difficulties or early awakening [67]	Superior sleep maintenance; minimal rebound after discontinuation [67]	Daytime somnolence; dizziness; nightmares [65]	Improved sleep maintenance [68]
Daridorexant	OX2R ≈ 0.23; OX1R ≈ 5.5; OX1R/OX2R = 24 [58]	~8 h [10]	25–50 mg [69]	Idorsia 2022	Difficulty initiating and/or maintaining sleep [59,70]	Patients requiring reduced residual effects [59]	Reduced next-day residual effects; stable plasma concentration profile [59]	Fatigue; headache; dry mouth [59,70]	↓ Daytime dysfunction [70]

Abbreviations: Ki, inhibition constant; t_1/2_, elimination half-life; LPS, latency to persistent sleep; WASO, wake after sleep onset; TST, total sleep time; ↑, increase; ↓, decrease.

**Table 2:** Structural design features and receptor selectivity of representative approved and investigational orexin receptor antagonists.

Drug	Structural Characteristics	Receptor Selectivity	Pharmacokinetic (PK) Characteristics
KHN707	Heterocyclic optimized scaffold [54]	Tendency toward OX2R selectivity [81]	Improved metabolic stability [81]
Fazamorexant	Flexible heteroaromatic scaffold [85]	Relative preference toward OX2R [84]	Optimized CNS exposure profile [84]

## Data Availability

No new data were created or analyzed in this study. Data sharing is not applicable to this article.

## Abbreviations

The following abbreviations are used in this manuscript:

CNS	central nervous system
COPD	chronic obstructive pulmonary disease
cryo-EM	cryo-electron microscopy
CYP3A4	Cytochrome P450 3A
Cys	cysteine residues
DORAs	Dual Orexin Receptor Antagonists
GABA	γ-aminobutyric acid
GPCR	G Protein-Coupled Receptor
LPS	Latency to Persistent Sleep
MDD	major depressive disorder
NREMS	non-rapid eye movement sleep
ORAs	Orexin Receptor Antagonists
OSA	Obstructive Sleep Apnea
OX1R	Orexin Receptor 1
OX2R	Orexin Receptor 2
PK	Pharmacokinetics
PLC	phospholipase C
REMS	rapid eye movement sleep
SAR	structure–activity relationship
SORA	Selective Orexin Receptor Antagonist
TST	Total Sleep Time
WASO	Wake After Sleep Onset
Z-drugs	non-benzodiazepine sedative–hypnotics
2-SORAs	selective orexin receptor 2 antagonists
1-SORAs	selective orexin receptor 1 antagonists
